# Supplementary material for: Human organoids for rapid validation of gene variants linked to cochlear malformations
Source: Hum Genet. 2025 Jan 9;144(4):375–89. doi: 10.1007/s00439-024-02723-9 (PMC12003500; doi:10.1007/s00439-024-02723-9)
Supplement: Supplementary file 1 — Supplementary Material 1 [file 439_2024_2723_MOESM1_ESM.docx]

| NAME | GUIDE SEQUENCE 5’-3' | GENOME | HDR DONOR SEQUENCE 5’-3' |
| --- | --- | --- | --- |
| *GREB1L* | TCTGGAAATTGTATCGGCTG | hg38 | TGGTTTTCCTGACTTCAGGGTTTTCTGGA  AATTGTATCGGCCGTGGAGAAAGAGGAT  TTCGATATTTCACGGAATTTTCCAA |
| *FGF3* | CGGGTCTTGAAGCCCCTGCG | hg38 | AACAGGGAGGACTTCTGTGTGCGGCGGG  TCTTGAAGCCCCCGCGGGGCCGGCCCTT  GCCGTTCACAGACACGTACCACAGT |
| *PBXIP1* | GGGCCTCAACAGCTCTGCCC | hg38 | CAGCCCTGGGGTGCCCGGTACTTGGGCC  TCAACAGCTCTGCTCAGGATGGCAGGGG  GTCATGGCTGTCCTTAGTCCCTTCCC |
| *GREB1L* | AGTGGTAGAGAGGGCAGAGA | | CCCCTTCCCAAGAGACGAAT |
| *FGF3* | AGTCAGAGTCAAGAGGCTGC | | TTTGGCACTGGACCGAACA |
| *PBXIP1* | CATGACGGAAGATGCCATCC | | CCCACCTCTTCTTCAGTGCT |

**Supplementary Table 1: Sequences of sgRNAs, HDR Templates, and Sanger Sequencing Primers Used for CRISPR-Cas9 Editing**

| **Target Sequence 5’-3'** | **PAM site** | **Score** | **#MM** | **Gene** | **Locus**  **GRCh38/hg38** | **Primer sequence 5’-3'** | **Mutation** |
| --- | --- | --- | --- | --- | --- | --- | --- |
| **FGF3** | | | | | | | |
| GGGTTCCTGAAGCCCCTGCA | GAG | 22 | 4 | - | Chr17:46891780 | AAACCTTCTCCCTCCCAAAG  CCTGTAATCCCAGCTACTCCA | No |
| CAGGGCATGAGGCCCCTGCG | TGG | 25 | 4 | - | Chr17:81689914 | AGTGTGGAGAACACGGGAAG  AACATGGCATCGCTCTCTTT | No |
| AGGGCCTTGAAGCCCCTGCT | GGG | 27 | 3 | - | ChrX:17659060 | AGGGCTGCTTCCTCTTGAA  CTTGTCCAGAGCCAAAGTCC | No |
| TGGG-CTTGAAGCCCCTGTG | AGG | 32 | 3 | - | Chr9:18055863 | CACGAGGAGTCGTAAGCACA  CAGTTCCTAAGGCCTCTTTCC | No |
| CAGGTCTGGAAGCCCCGGCG | CAG | 32 | 3 | *DISP3* | Chr1:11534485 | ACACACGCACCACTCCATT  TTTAGGTGGCCAAAGTCCAG | No |
| **PBXIP1** | | | | | | | |
| CTGCCTCCACAGCTCTGCCC | CAG | 4 | 3 | - | Chr20:37388157 | CTGAGTGCTTGAAGTTGCGT  GTTCAAGCGATCCTCCTGC | No |
| TGCCCTC-ACAGCTCTGCCC | TGG | 5 | 3 | - | Chr12:4827719 | GTTTTGGCCGTTTCAAGCAC  ACCGAGTTCCAACACTGAGT | No |
| GGCTCTCAGCAGCTCTGCCC | GGG | 7 | 3 | - | Chr10:95291214 | CTGGAGGTCTATCTGCTGGG  CGGGCAAGTCTTCTCACCTA | No |
| GGGCTCTCAGCAGCTCTGCCC | GGG | 10 | 2 | - | Chr10:95291213 | CTGGAGGTCTATCTGCTGGG  CGGGCAAGTCTTCTCACCTA | No |
| GGGCGTC-ACAGCTCTACCC | TGG | 10 | 3 | - | Chr12:34265005 | GGTGTGCCTCCATCTTCTCTCAG  CAGCACTCACGGATTTC | No |
| **GREB1L** | | | | | | | |
| ACTGAAAATTGTATCAGCTG | GGG | 20 | 3 | - | Chr15:79626682 | AACCTCTCTTGACTCTGGGT  GGTGTTCACTATACAGACATCCT | No |
| TCAGGAAATTGCATCAGCTG | CAG | 32 | 3 | - | Chr5:14532526 | AAAACCAAGACATCACCCCG  AGAGATAGAGTTGCTCCACCTC | No |
| TATGGATATTGTATCAGCTG | GAG | 34 | 3 | - | Chr11:93256237 | TGCCCCAGGTGTAATCAAATG  TGTCACAAGCCTTTCTACATTTG | No |
| TCAGGACGTTGTATCGACTG | AGG | 39 | 4 | *PLA2G4F* | Chr15:42142060 | CGCTGTGAAATGAGTGCTGT  TGGGTCTCAGCGTTCAGAT | No |
| TCTAAAAATTGTAT-GGCTG | GGG | 44 | 3 | - | Chr11:124808824 | TGCACAAGAATCTCAAAACTCAA  CCTCTCACCATTTCCAGGCA | No |

**Supplementary Table 2:** Off-target sequencing analysis. The table presents the off-target analysis for CRISPR-Cas9 editing in our study. The MM (mismatch) score reflects nucleotide differences between the CRISPR guide RNA and potential off-target sites, with lower scores indicating higher off-target risk. The associated PAM site, ideally NGG, is also critical for CRISPR activity. The "Score" is a composite metric considering mismatch count and position relative to the PAM site, predicting CRISPR-Cas9 activity at off-target sites. Nucleotide Highlighted with Red show mismatches (MM).

| Family ID | Phenotype | Gene | Variant Characteristics | Frequency | | Pathogenicity Scores | | | ACMG Criteria | ACMG Classification and scores^d^ | Improved ACMG Classification and scores after study |
| --- | --- | --- | --- | --- | --- | --- | --- | --- | --- | --- | --- |
|  |  |  |  | gnomAD  (v4) | Internal controls (1612 samples) | REVEL^a^ | MAVERICK^b^ | AlphaMissense^c^ |  |  |  |
| Family 1 | Bilateral Michel Aplasia, SNHL | *FGF3* | NM_005247.4  c.493A>G  p.Arg165Gly | N/A | N/A | 0.6 | 0.16 | 0.8 | PM2_sup,PP3_mod, PM3_sup | VUS  (4) | PM2_sup,PP3_mod, PM3_sup  PS3_sup^e^  VUS (5) |
| Family 2 | Unilateral Common Cavity, SNHL | *GREB1L* | NM_001142966.3  c.556T>C  p.Cys186Arg | N/A | N/A | 0.8 | 0.9 | 1 | PM2_sup, PP3_mod, PS2_mod | VUS  (5) | PM2_sup, PP3_mod, PS2_mod, PS3_sup  LP (7) |
| Family 3 | Bilateral Cochlear Aplasia, SNHL | *PBXIP1* | NM_020524.4  c.1722G>A  p.Trp574* | N/A | N/A | N/A | 1 | N/A | PVS1_sup, PM2_mod | VUS  (3) | PVS1_strong, PM2_mod, PS3_sup  LP (8) |

**Supplementary Table 3:** Details of phenotypes and identified variants

**Abbreviations**: **SNHL**; Sensorineural Hearing Loss, **gnomAD**; Genome Aggregation Database, **N/A**; Not Available, **REVEL**; Rare Exome Variant Ensemble Learner, **MAVERICK**; Mendelian Approach to Variant Effect pRedICtion built-in Keras, **sup**; supporting, **mod**; moderate, **VUS**; Variant of Uncertain Significance

All the genomic coordinates are from GRCh37/hg19 reference assembly.

^a^PMID: 27666373, ^b^PMID: 37443090, ^c^ PMID: 37733863, ^d^PMID: 32720330, ^e^PMID: 31892348

| Chromosomal location (hg19) | Potential variants to be tested | MAF  (gnomAD v4) | Pathogenicity scores | | | OMIM ID |
| --- | --- | --- | --- | --- | --- | --- |
|  |  |  | **REVEL** | **AM** | **MAVERICK** |  |
| 1 : 84,864,256-90,179,532 | Chr1:154918428  *PBXIP1* (NM_020524.4)  c.1722G>A; p.Trp574* | Absent | N/A | N/A | 0.9 | N/A |
| 1 : 109,268,573-118,644,430 | N/A | N/A | N/A | | | N/A |
| 1 : 149,906,413- 158,813,819 | Chr1:153748639  *SLC27A3* (NM_024330.4)  c.666A>C; p.Pro222Pro | Absent | N/A | N/A | 0.4 | N/A |
| 5 : 52,096,889-56,778,103 | N/A | N/A | N/A | | | N/A |
| 5 : 70,308,251-73,992,881 |  |  |  |  |  |  |
| 5 : 112,162,854-114,462,355 |  |  |  |  |  |  |
| 10 : 97,141,523-105,824,333 | Chr10:101474456  *COX15* (NM_078470.5)  c.1121C>T; p.Thr374Met | 0.002 | 0.9 | 0.7 | 0.8 | 603646 |
| 15 : 62,456,358-68,649,587 | Chr15:62762350  *TLN2* (NM_015059.2)  c.4858C>T; p. Arg1620Cys | 0.004 | 0.3 | 0.4 | 0.0 | N/A |
| 17 : 617,869-9,586,165 | Chr17:2036859  *DPH1* (NM_001383.4)  c.598G>A; p.Glu200Lys | Absent | 0.6 | 0.1 | 0.8 | 603527 |
| 17 : 71,746,796-74,733,099 | N/A | N/A | N/A | N/A | N/A | N/A |
| 21 : 30,339,120-36,080,290 | Chr21:34962133  *CRYZL1* (NM_145858.3)  c.1045T>C; p. Phe349Leu | Absent | 0.06 | 0.7 | 0.2 | N/A |

**Supplementary Table 4:** Homozygous regions and variants present in the proband of family 3. (All the genomic coordinates are from GRCh37/hg19 reference assembly**.)**

**MAF;** Minor allele frequency, **N/A;** Not Applicable, **REVEL;** Rare Exome Variant Ensemble Learner, **AM;** AlphaMissense, **MAVERICK;** Mendelian Approach to Variant Effect prediction built in Keras


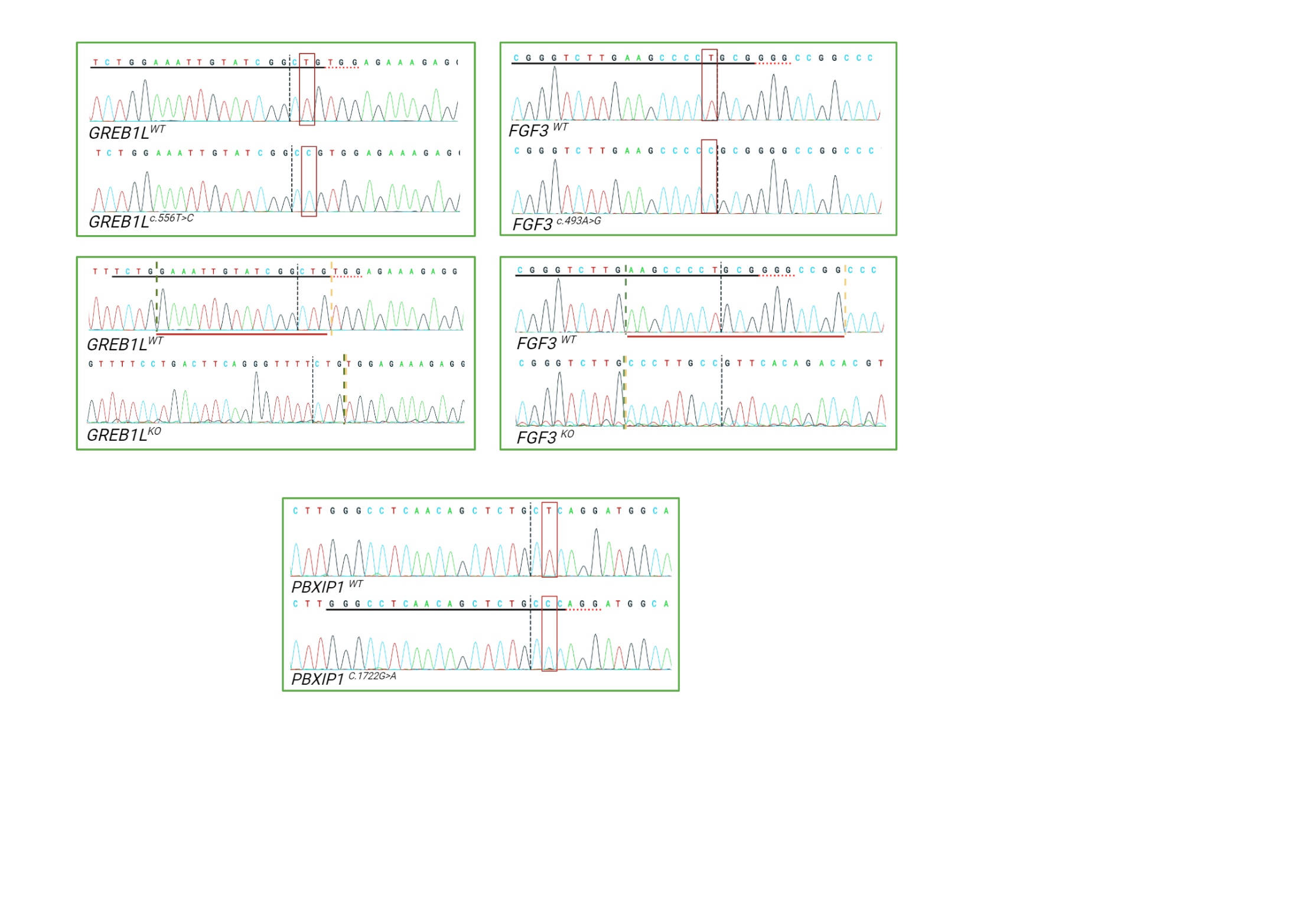


**Figure S1:** Confirmation of Variant-Bearing iPSCs via Sanger Sequencing. The sequence alignment shows the sgRNA target site, indicated by the (_) symbol, with the Cas9-induced double-stranded break site marked by a vertical dotted line near the GG PAM site. This analysis confirms the presence of the intended genetic variant in the iPSCs, as indicated by the sequence alterations..

**Figure S2: Inner ear imaging details from available probands.** **(A)** CT scans for proband **(**II:1) from family 2 showing absence of the cochlea with a dilated vestibule (curved white arrow) on the right side (common cavity). IAC is narrow on the right side (white arrow). Normal cochlea (black arrow) and IAC (curved black arrow) is visible on the left side; **(B)** Proband from family 3 (II:1) with bilateral cochlear aplasia. Contiguous axial CT slices reveal bilateral cochlear aplasia with only sclerotic bone (arrow) and deformed cystic vestibule (curved arrow).


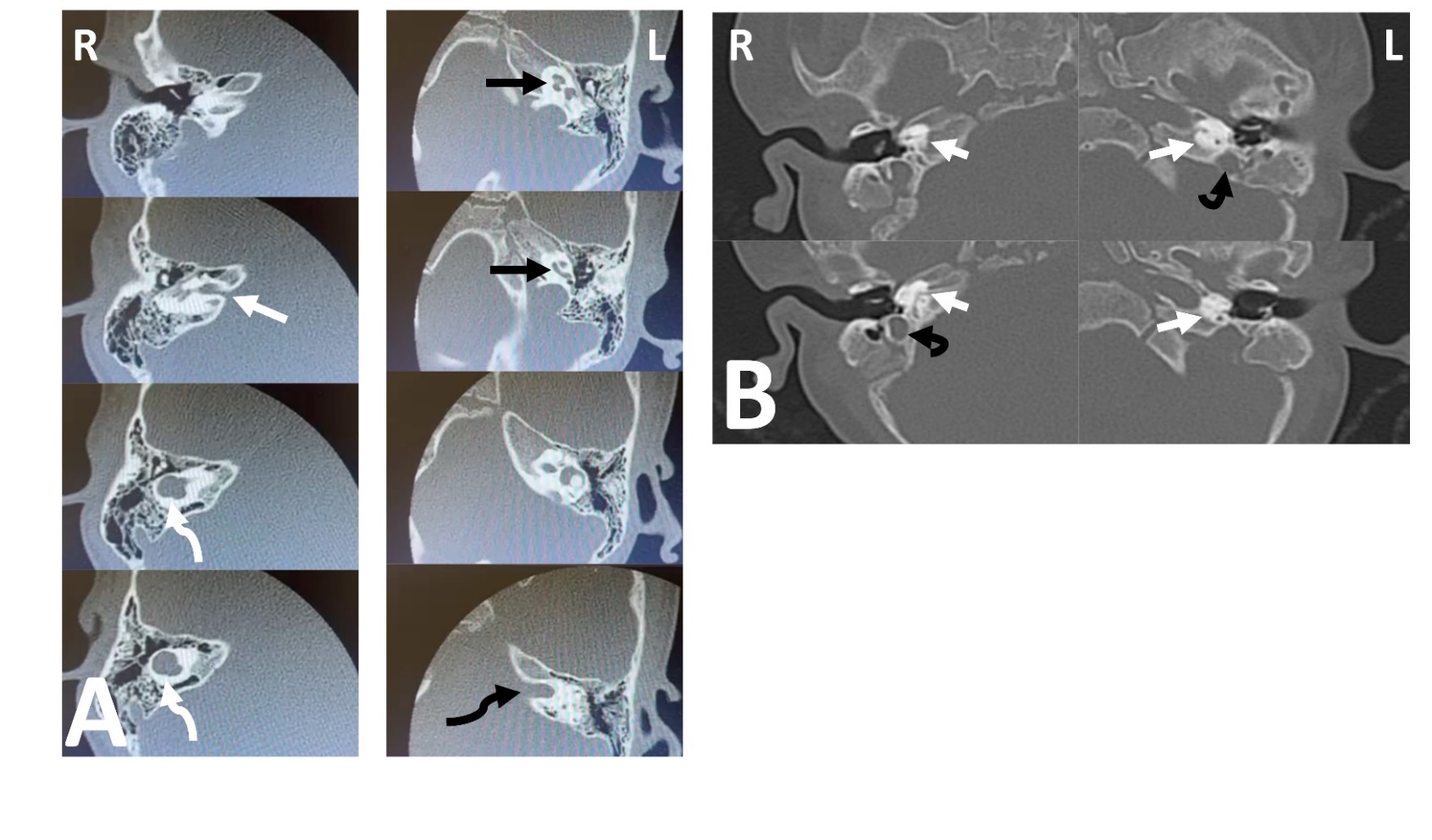


**
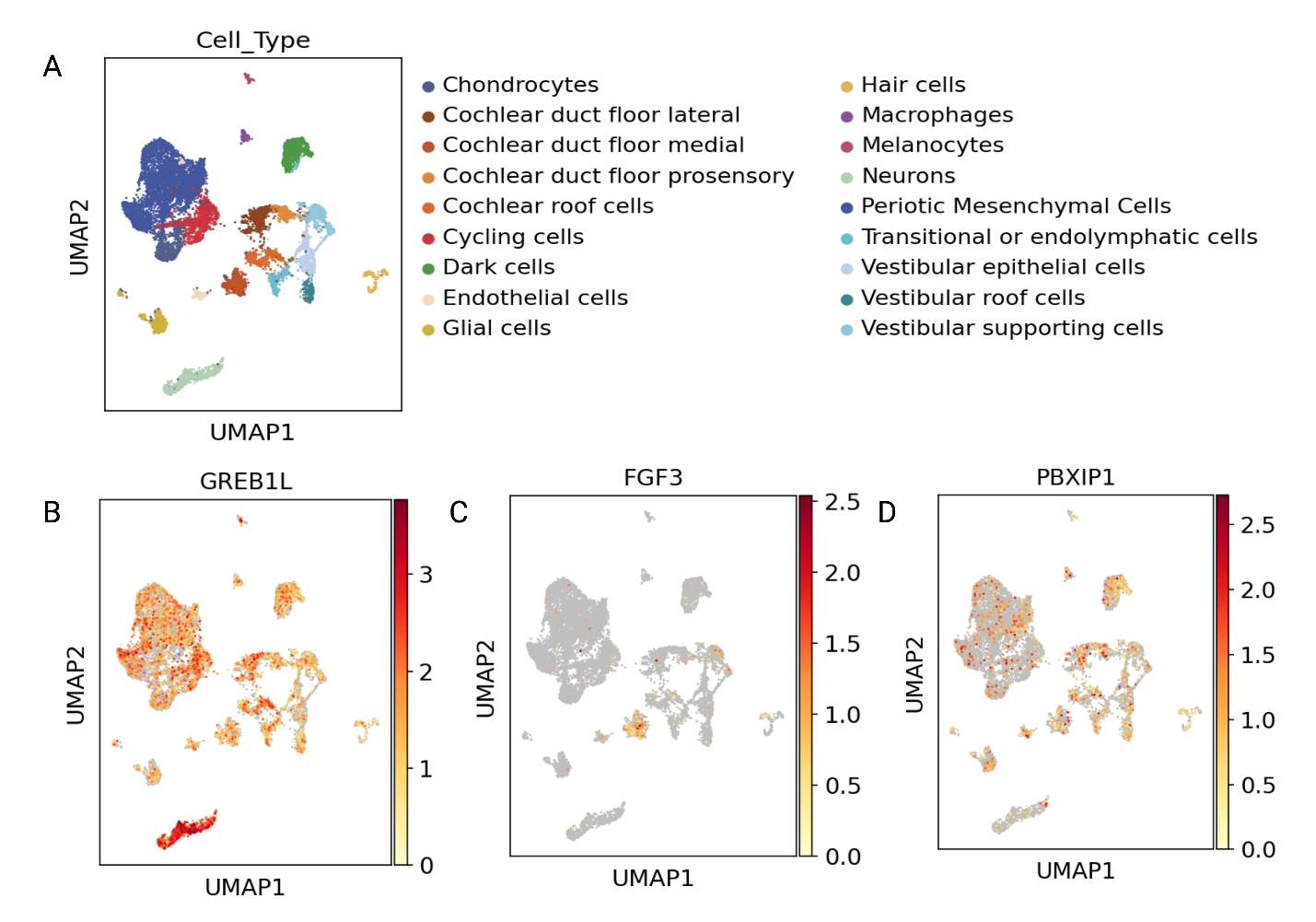
**

**Figure S3:** U-map Plots of snRNA-seq-Based Expression of GREB1L, FGF3, and PBXIP1 in Different Cell Clusters of the Developing Human Cochlea (Data from <https://umgear.org/>). (A) Localization of distinct cell types within the cochlear tissue, with cluster identities highlighted. (B) Gene-specific expression patterns for GREB1L, FGF3, and PBXIP1, showing differential expression across cell clusters


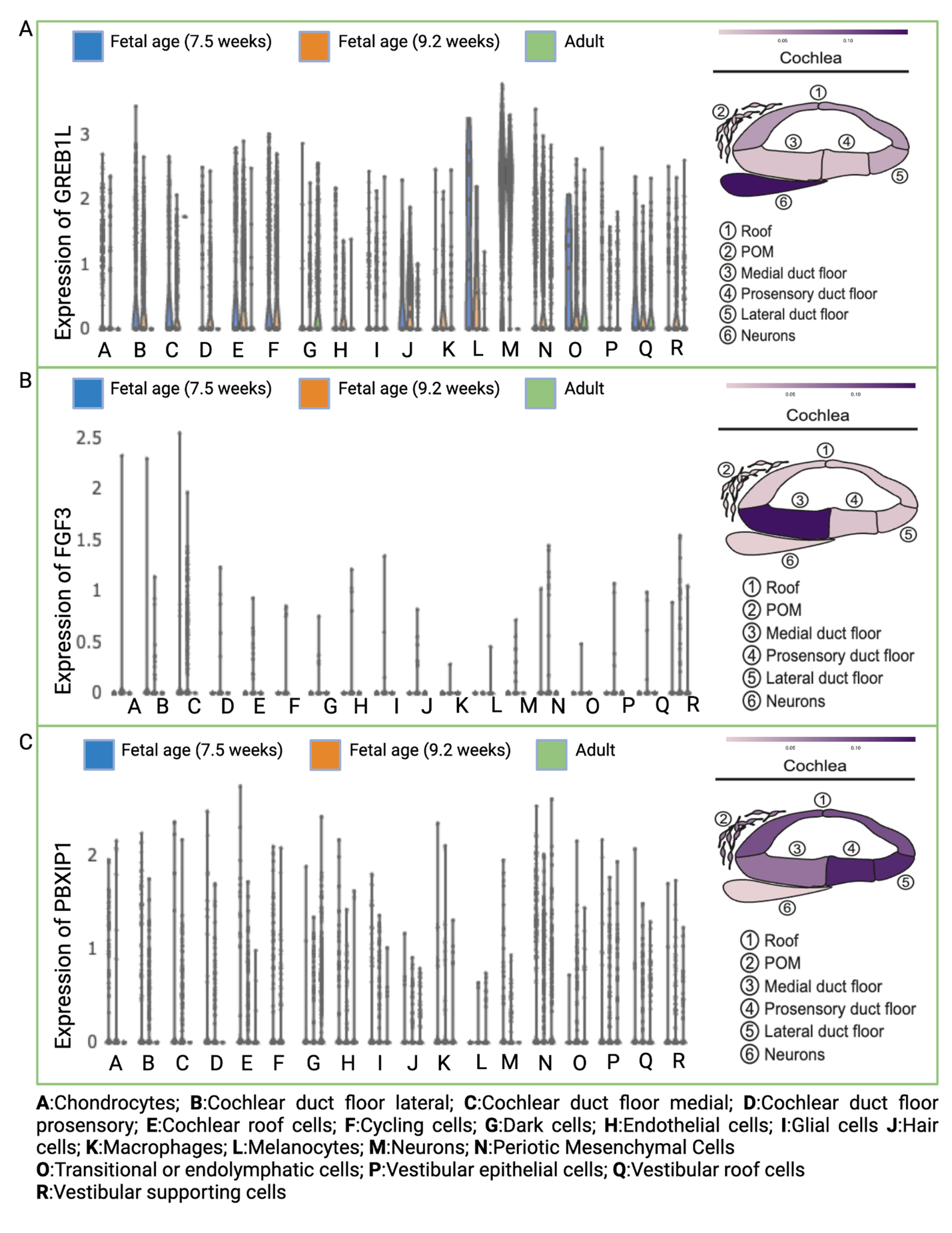
 **Figure S4:** snRNA-seq profile of *GREB1L*, *FGF3,* and *PBXIP1* expression in human cochlea at different ages and spatial localization (<https://umgear.org/>).


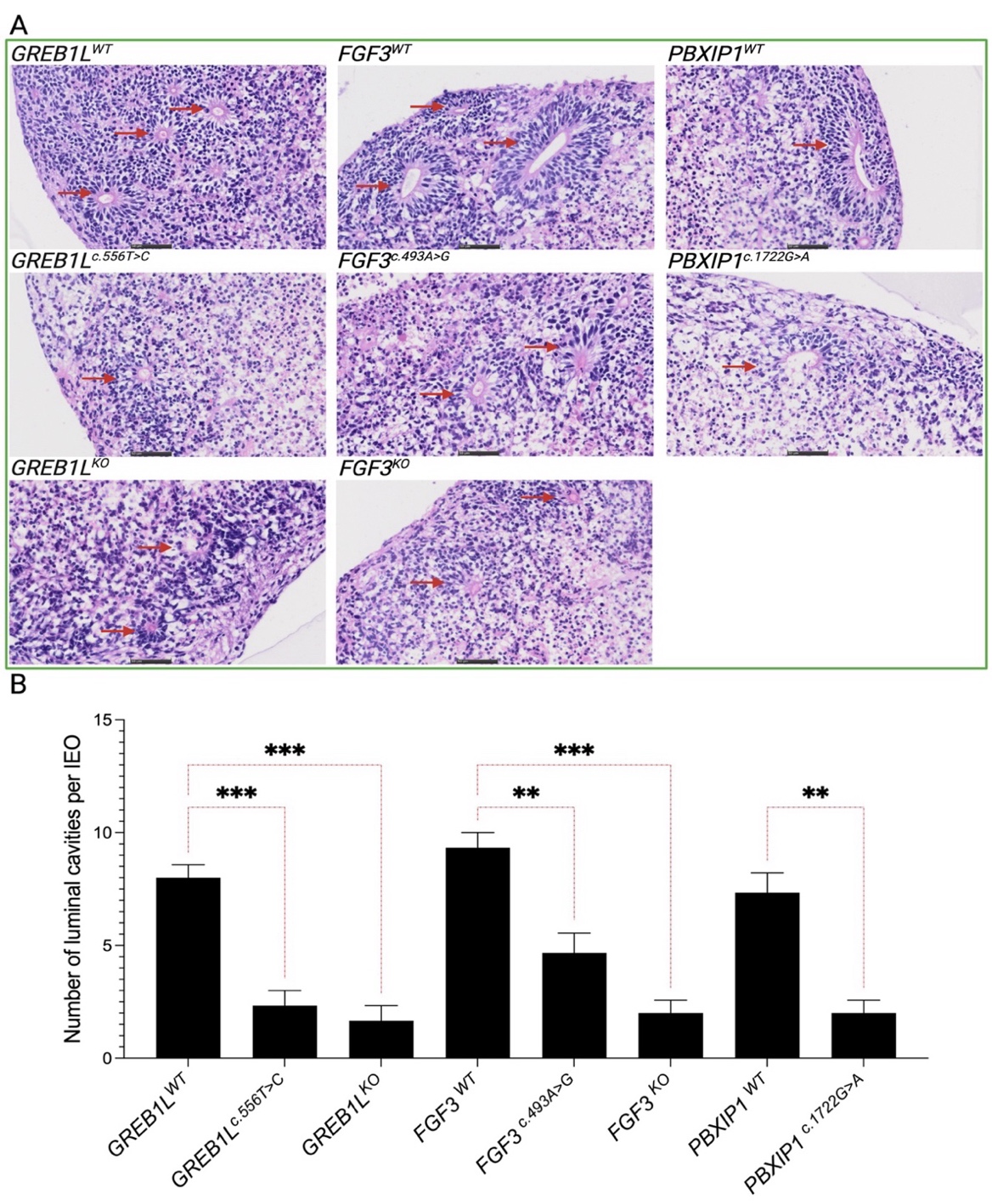


**Figure S5:** Representative images and statistical analysis of day 35 IEO (n=3) showing the effect of *FGF3^KO^*, *FGF3^c.493A>G^*, *GREB1L^KO^*, *GREB1L ^c.556T>C^*, and *PBXIP1^c.1722G>A^* on the numbers of luminal spaces and cell confluence around luminal spaces/cavities (red arrows) that mature into hair cell-like cells bearing cavities**.** The results are expressed as Mean ±SEM, and the statistical difference of p ≤0.05 was considered significant; the significant differences are marked with (*).


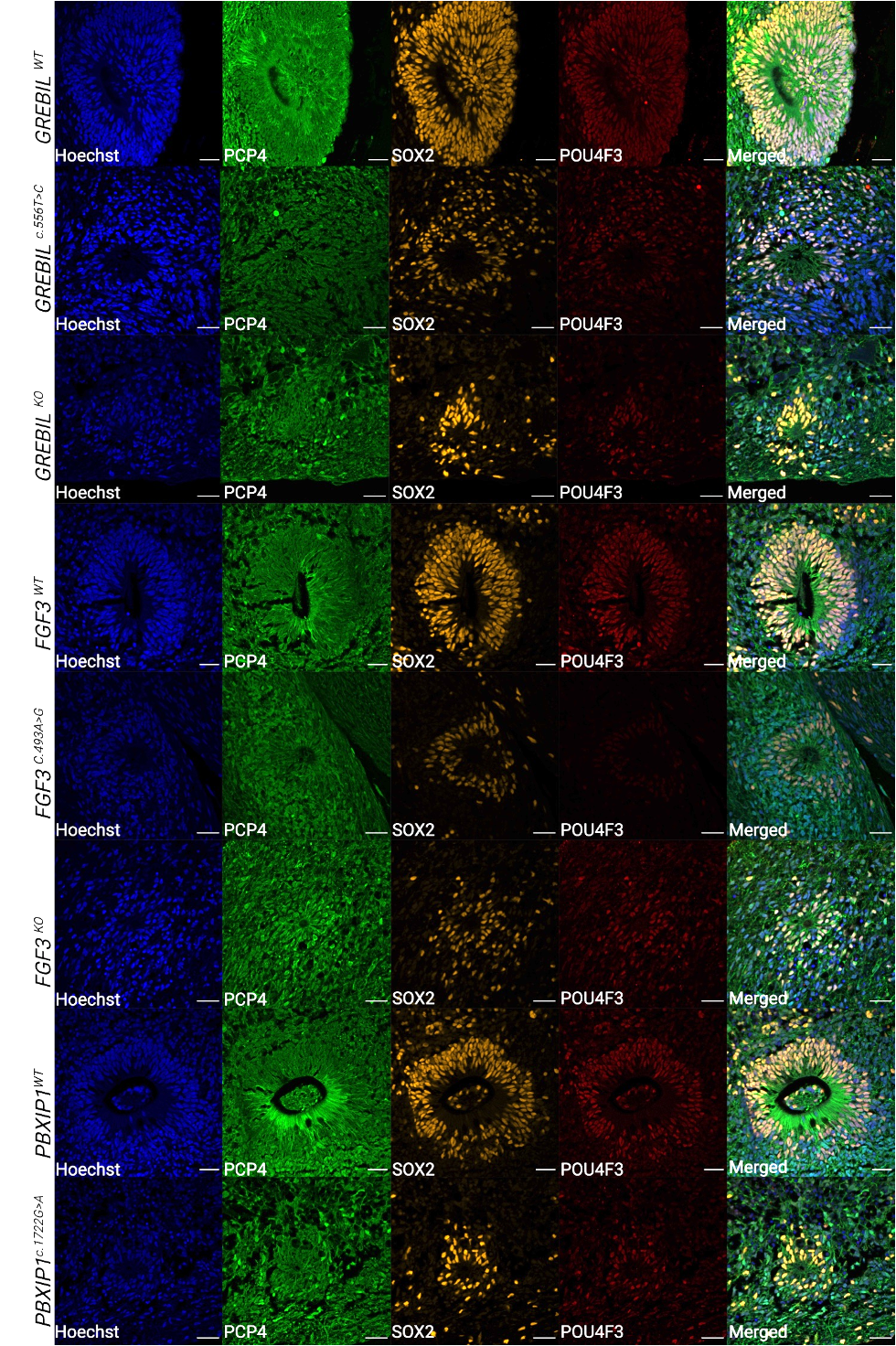


**Figure S6:** Inner ear hair cell population confirmation by PCP4, POU4F3 (BRN3c), and SOX2 immunohistochemistry. The images were acquired using Carl Zeiss LSM 710 confocal microscope.


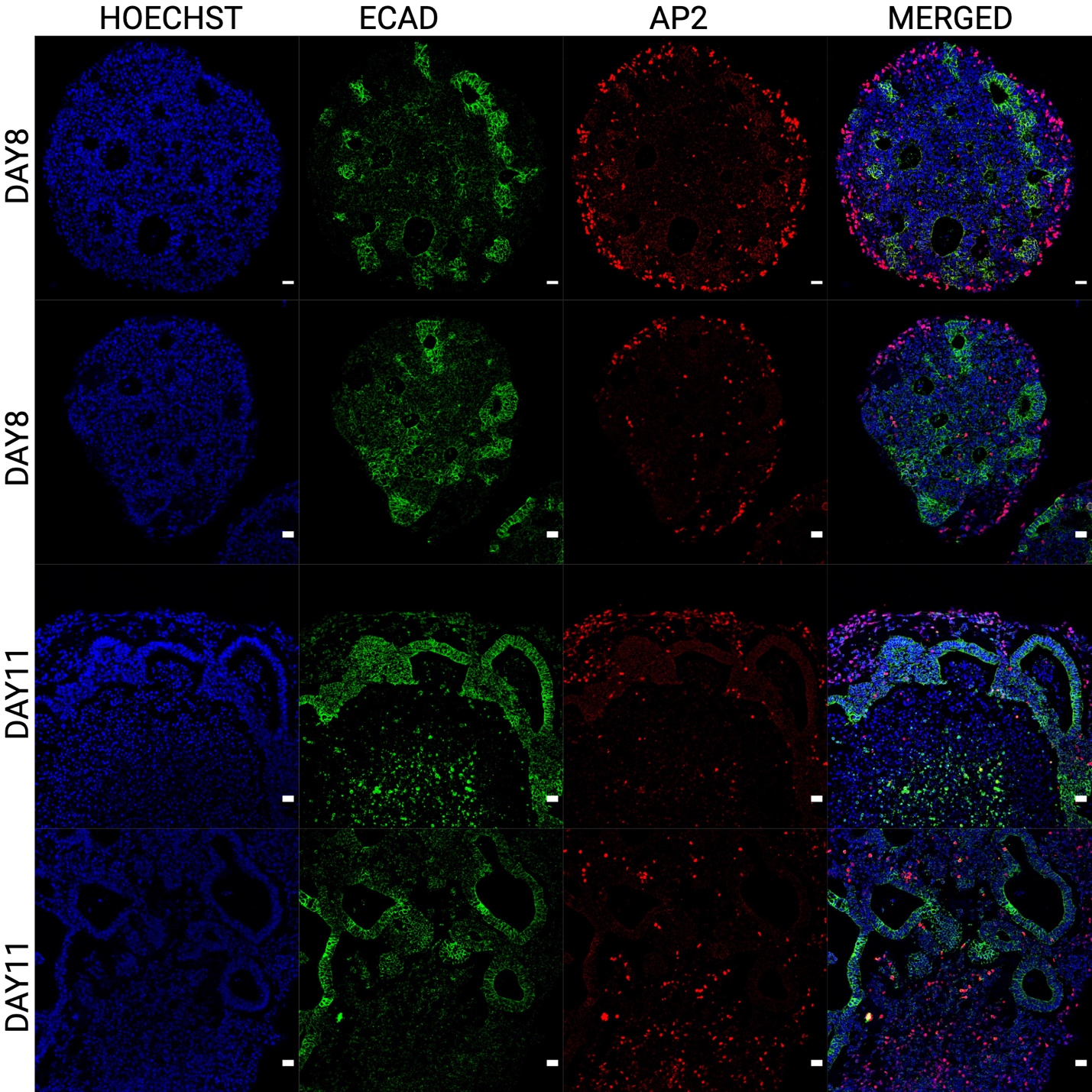


**Figure S7:** Representative images of day 8 and day 11 inner ear organoids derived from the control line ASE9203. Immunohistochemistry was done to confirm CDH1 and AP2expression.


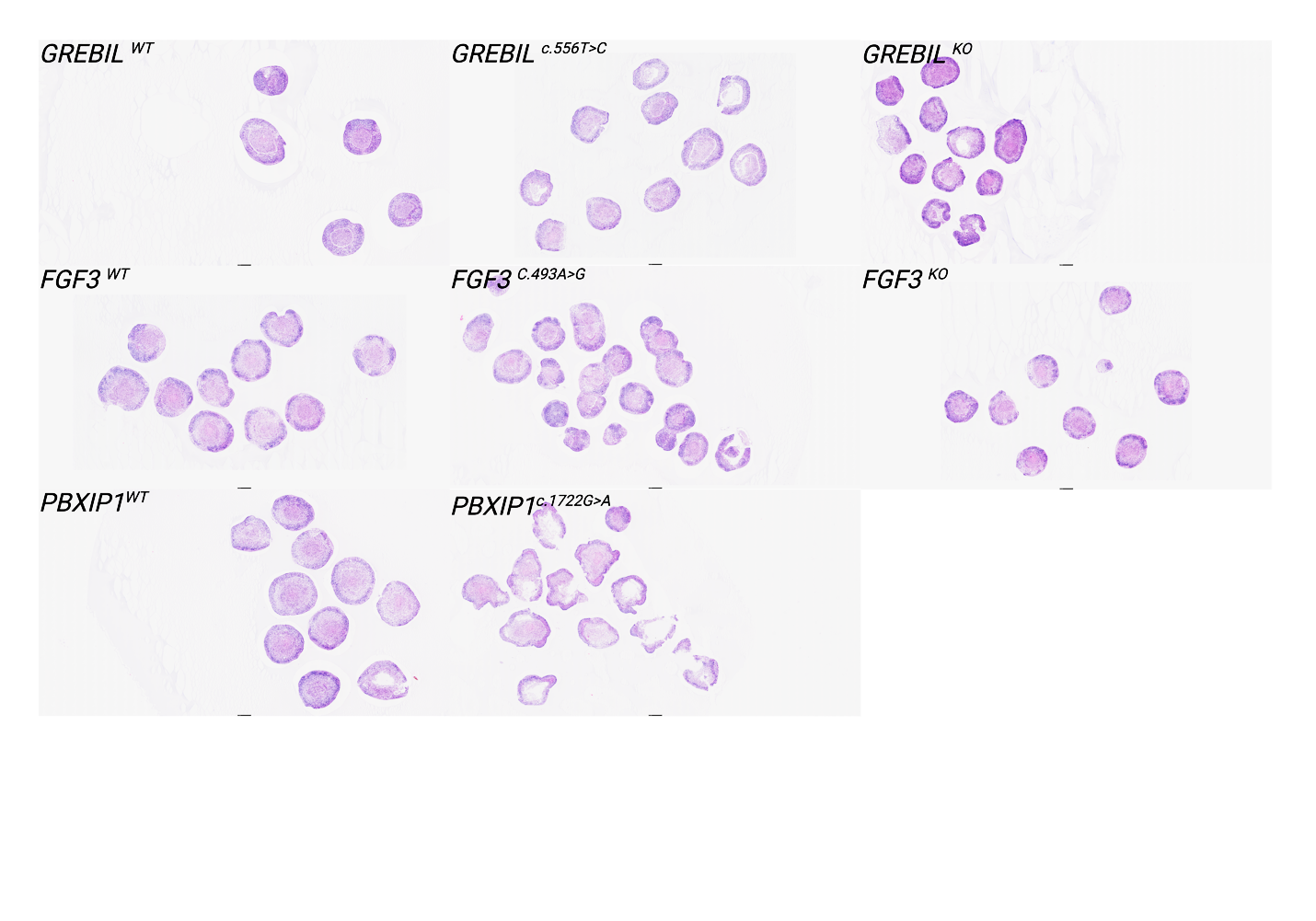


**Figure S8**: Images of H&E stained zoomed-out slides of inner ear organoids. The images provide an overview of the morphological structure of the day 35 organoids, highlighting key features and cellular organization under low magnification.


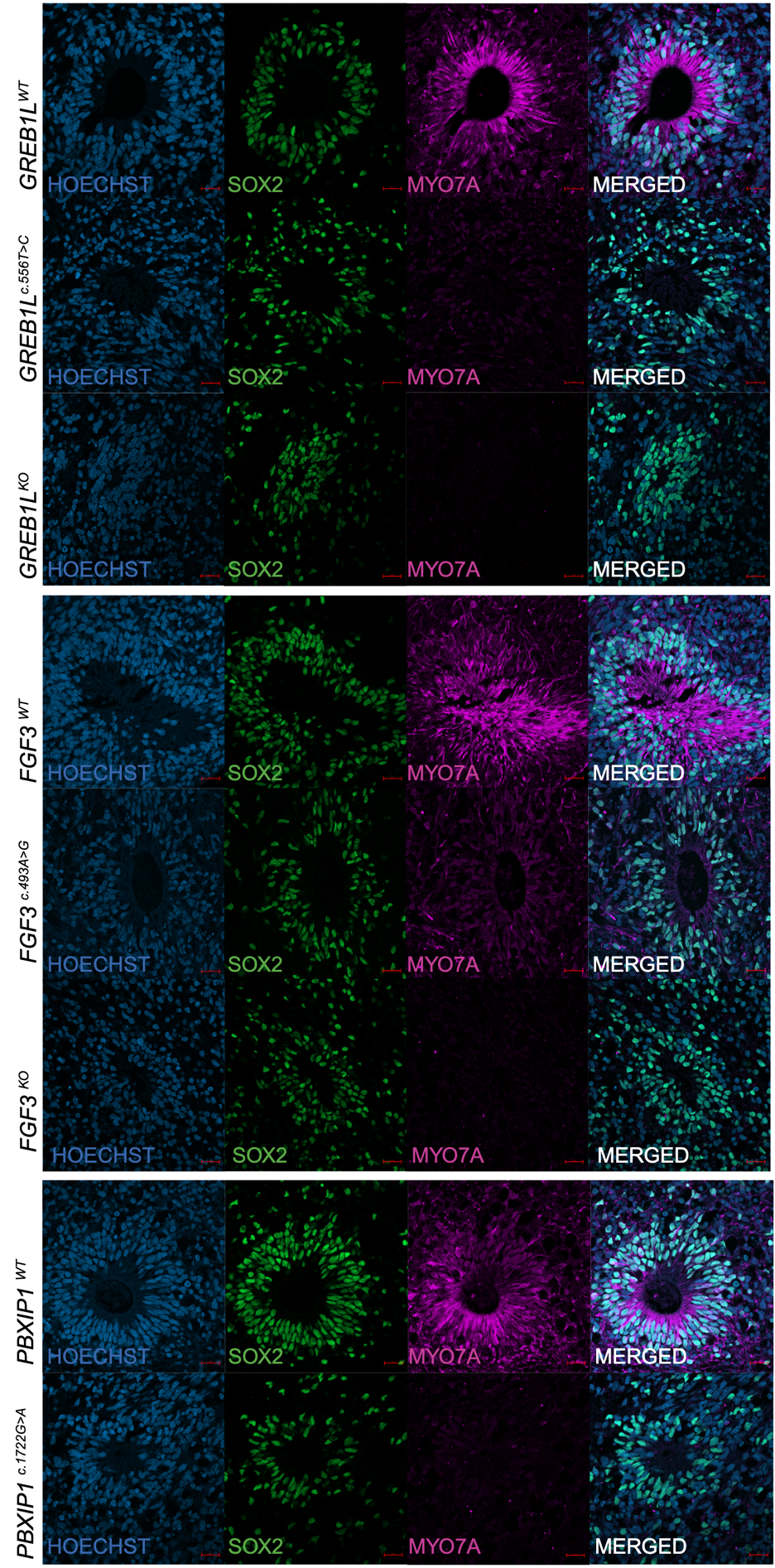


**Figure S9**: Full Panel of Images for Figure 4, Displaying Individual Channels and Composite Views. Each panel presents the individual fluorescence channels for the relevant markers, as well as composite images combining all channels to show co-localization patterns and cellular structures
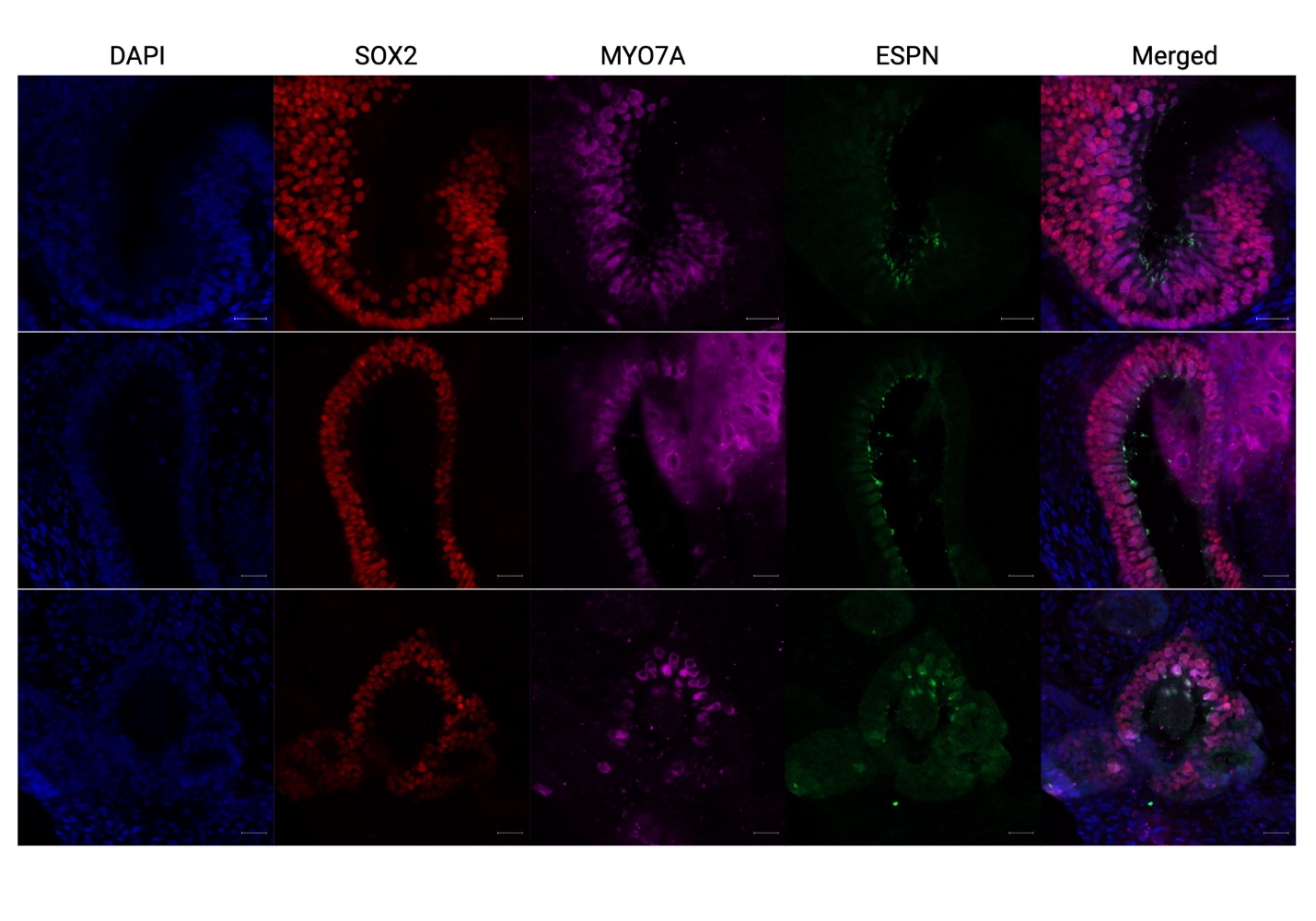


**Figure S10:** Representative Images of Control Line Staining for Hair Cell-Like Cell Bundles. The images show co-localization of DAPI (Blue) for nuclei, SOX2 (Red) marking progenitor cells, MYO7A (Magenta) as a marker for hair cell differentiation, and ESPN (Green) highlighting stereociliary components. These staining patterns are indicative of hair cell-like cell bundles in the control line.
